# Chemical Characterization and Chemotaxonomic Significance of Essential Oil Constituents of *Matricaria aurea* Grown in Two Different Agro-Climatic Conditions

**DOI:** 10.3390/plants12203553

**Published:** 2023-10-12

**Authors:** Merajuddin Khan, Mujeeb Khan, Eman Alshareef, Shatha Ibrahim Alaqeel, Hamad Z. Alkhathlan

**Affiliations:** 1 Department of Chemistry, College of Science, King Saud University, P.O. Box 2455, Riyadh 11451, Saudi Arabia; kmujeeb@ksu.edu.sa; 2 Department of Chemistry, College of Science, King Saud University (034), Riyadh 11495, Saudi Arabia; 438203607@student.ksu.edu.sa (E.A.); shalaqeel@ksu.edu.sa (S.I.A.)

**Keywords:** essential oils, *Matricaria aurea*, chemical profiling, volatiles, phytoconstituents

## Abstract

A comprehensive study on chemical characterization of essential oil (EO) constituents of a rarely explored plant species (*Matricaria aurea*) of the Asteraceae family grown in Saudi Arabia and Jordan was carried out. Analyses were conducted employing gas chromatographic approaches such as GC-MS, GC-FID, and Co-GC, as well as RT, LRI determination, and database and literature comparisons, on two diverse stationary phase columns, which led to the identification of a total of 135 constituents from both EOs. Oxygenated sesquiterpenes were found to be the most predominant chemical class of Saudi *M. aurea* EOs, in which *α*-bisabolol (27.8%), *γ*-gurjunenepoxide (21.7%), (*E*, *E*)-*α*-farnesene (16.3%), and *cis*-spiroether (7.5%) were present as major components. In contrast, the most dominant chemical class of Jordanian *M. aurea* oil was found to be sesquiterpene hydrocarbons, where (*E*, *E*)-*α*-farnesene (50.2%), *γ*-gurjunenepoxide (8.5%), (*E*)-*β*-farnesene (8.1%), and (*Z*, *E*)-*α*-farnesene (4.4%) were detected as chief constituents. It is interesting to mention here that Saudi and Jordanian *M. aurea* EOs showed quite interesting chemical compositions and were found to have different chemotypes when compared to previously reported *M. aurea* EO compositions.

## 1. Introduction

Currently, the increasing threats of contagious diseases and epidemics have forced scientists to explore different natural resources, such as medicinal plants and other marine organisms, for the development of novel pharmaceutics based on traditional knowledge [1,2,3]. As of now, a variety of plant materials have been significantly explored and applied as precursors in different pharmaceutical, fragrance, and cosmetic industries to derive novel drugs, perfumes, and other applied ingredients for different medicinal applications [4,5]. Over the years, despite significant success in using synthetic substances in the development of novel pharmaceutics, natural products, and particularly plant-derived materials, are still regarded as reliable sources of medicines and other applied materials, and are thus very popular in different industries [6,7,8]. Indeed, due to the recent advancements in the techniques of synthesis and identification of phytochemicals, an enormous number of phytoconstituents have been extracted from plants and tested for their medicinal potential by utilizing modern methodologies and traditional knowledge [9,10]. Among these medicinal plants, *Matricaria aurea*, which is an effective herb of the genus *Matricaria*, is an indigenous drug of the Kingdom of Saudi Arabia, and is largely known for its therapeutic potential and considered a promising source of antimicrobials and antioxidant agents. This plant exhibits various resemblances with *M. chamomilla* (chamomile), particularly in terms of the composition of its phytoconstituents of essential oils, such as flavones and flavonoids [11]. Moreover, the traditional applications of *M. aurea* are also similar to those of the main species (chamomile), and thus it is extensively applied and globally considered to be one of the ancient medicinal plants [12].

This crucial medicinal (*M. aurea*) plant is a member of the Asteraceae family, and is typically found in Saudi Arabia and in several other parts of the world. It has been widely applied in folk medicines for several diseases including cough, spasmodic, asthma, flatulence, common cold, and influenza [13]. In addition to these, this plant is also known to exhibit analgesic and anti-inflammatory activities [14]. So far, different types of phytoconstituents, including coumarins and quercetins, caffeic acid, apigenin-7-*O*-glucoside, umbelliferone, and naringenin, have been extracted from different species of *Matricaria*. Still, the detailed exploration of the phytochemical profile of *M. aurea* has been rarely performed, except in a few studies, which have superficially highlighted types of phytomolecules present in the species. For example, in our previous study, we prepared different extracts of *M. aurea*, which were explored for their anticorrosive properties against mild steel (MS) in corrosive media (1.0 M HCl). Among the different studied extracts, including water, *n*-hexane, and methanol, the methanolic extract demonstrated superior anticorrosive property, and was chosen for further detailed analysis to determine the active phytomolecules responsible for the anticorrosive action. This detailed analysis led to the discovery of a novel green corrosion inhibitor named apigetrin [15]. Subsequently, in another study, Ahmad et al. studied the antibacterial efficacy of the ethanolic extract of *M. aurea,* which was tested against a variety of clinical isolates [12]. Similarly, the same group further explored the remarkable medicinal properties of *M. aurea* by testing the anticancer properties of the plant against human breast adenocarcinoma (MCF-7) and other cell lines [16]. Apart from these, and a few other studies, the aforementioned plant has not been explored appropriately according to its medicinal potential; in particular, the plant species of *M. aurea* from Saudi Arabia has been rarely explored.

It is worth mentioning that species of the same plant growing in different regions of the world generally exhibit vast chemical diversity, which is typically attributed to the presence of varying chemotypes. For example, the comparative analysis of the phytomolecules of the essential oils of the leaves and stems of *Achillea fragrantissima* of Saudi Arabia has revealed the presence of different types of major constituents when compared to the essential oils of the same plant grown in other regions of the world, such as Egypt, Jordon, and Yemen [17]. Generally, this type of chemical diversity can be attributed to different factors, such as genetic variations and ecological and environmental factors [18]. In addition, different atmospheric conditions, such as radiation levels, climatic conditions, temperature, and photoperiod, may also exert significant effects on the quantity and quality of the phytoconstituents [19,20]. Indeed, these types of chemical diversities (specific variety of the chemicals produced by the plants) have been effectively utilized for the classification of the plants. This is referred to as chemotaxonomic classification [21], which is a modern strategy used to classify the plants. For example, *A. fragrantissima* grown in different regions of the same country (Egypt) has exhibited varying chemotypes; a plant of the Sinai region showed *cis*-thujone as a major component, while plants grown in Saint Catherine and Sharkia possessed *α*-thujone and santolina alcohol as their lead component, respectively [17,22,23,24]. In addition, *A. fragrantissima* obtained from different regions of Jordan, namely Mafraq and Amman, have exhibited the presence of artemisia ketone and *α*-thujone as major components, respectively [25,26]. Similarly, *Plectranthus cylindraceus* grown in different parts of the world has exhibited various chemotypes; for instance, *P. cylindraceus* from Oman showed carvacrol as a major component, while the same plants grown in Yemen, Ethiopia, and Saudi Arabia possessed thymol, camphor, and patchouli alcohol as the most dominant compounds, respectively [27,28,29,30]. Therefore, chemotaxonomic evaluation of *M. aurea* grown in different regions of the world may reveal interesting information, and, to the best of our knowledge, chemical characterization of *M. aurea* grown in different agro-climatic conditions has not been done yet. Moreover, as discussed earlier, *M. aurea* has wide applications in traditional medicine, but despite its vast medicinal potential, it has been relatively less explored. Thus, herein, the essential oils of the plant species *M. aurea*, which were grown in Saudi Arabia and Jordan, were subjected to extraction and analyzed in detail using GC approaches such as GC-MS, GC-FID, and Co-GC, and RT and LRI determination techniques.

## 2. Results and Discussions

In order to analyze and compare the phytochemical profiles of both the essential oils of *M. aurea* from Saudi Arabia and Jordan, the essential oils of the plants were isolated using a conventional hydro-distillation process, which was performed for three hours in a Clevenger-type apparatus [31]. At the end of the extraction process, light-yellow-colored oils from both the plant materials were generated at the yields of 0.03% and 0.05%, respectively, which were measured as per the fresh weight of the plant materials. The chemical characterization of the extracted essential oils was carried out by applying GC-MS (gas chromatography–mass spectrometry) and GC-FID (gas chromatography–flame ionization detector) techniques on two different stationary phase (nonpolar and polar) columns. The GC analysis indicated the presence of 135 phytochemical constituents in the essential oils of both the plants from Saudi Arabia and Jordan. Out of these 135 identified constituents, only 56 phytomolecules were found to be present in both the essential oils, while 62 compounds were specific to the plant from Jordan and only 17 constituents were only associated with the essential oil of Saudi *M. aurea*. Notably, most of the specific phytochemical constituents of the Jordanian species were present in very minute quantities, i.e., 0.1 to 0.3%. The respective quantities of all the determined phytochemicals from both oils are presented in the form of a table (Table 1) based on their order of elution on a HP-5MS column.

As per the information given in Table 1, sesquiterpene hydrocarbons were present in the largest amount in Jordanian species, whereas the Saudi plant sample was mostly dominated by oxygenated sesquiterpenes.

For instance, the essential oil of Jordanian plant consists of 66.4% sesquiterpene hydrocarbons, while the Saudi species demonstrated the occurrence of 58.2% oxygenated sesquiterpenes. Notably, the sesquiterpene hydrocarbons and their oxygenated derivatives were dominant in both the essential oils; however, their amounts were different, i.e., the sesquiterpene hydrocarbons were present in 24.0% of the total contents in Saudi plant, whereas only 18.5% of oxygenated sesquiterpenes were present in the Jordanian species. On the second position, the Saudi species consisted of 7.7% polyacetylenic, while a similar group of compounds was found in the amount of 4.0% in the Jordanian species. It is worth mentioning that, in both the species, oxygenated aliphatic hydrocarbons were detected in a distant third position, and were present in almost the same quantity, i.e., 4.0% and 3.9% oxygenated aliphatic hydrocarbons in the Saudi and Jordanian plants, respectively. After these three major kinds of phytoconstituents, which were present in relatively large quantities, oxygenated monoterpenes (2.3% and 1.7%), monoterpene hydrocarbons (0.3% and 0.4%), aliphatic hydrocarbons (0.7% and 0.9%), diterpenoids (1.3% and 1.6%), and aromatics (1.0% and 1.3%) were also present in notable quantities in the Saudi and Jordanian *M. aurea*, respectively. In addition to these groups of compounds, other components were individually detected in miniscule amounts, but together they were significant, amounting to between 11 and 15% in both species. The total percentage of the identified compounds was found to be 98.3% and 98.7% in Saudi and Jordanian species, respectively.

**Figure 1:**
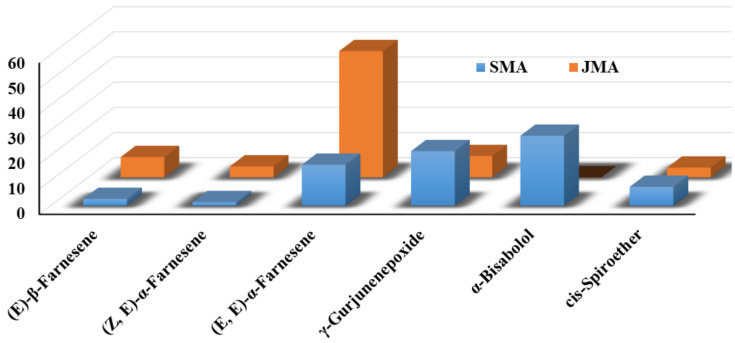
Comparison of major components of Saudi and Jordanian *M. aurea* essential oils.

**Figure 2:**
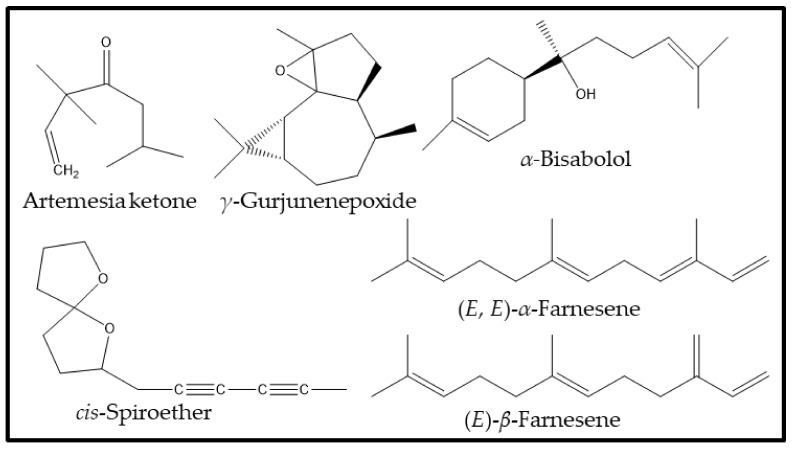
Chemical structure of the most dominant compounds from *M. aurea* essential oils.

There were only a few compounds that heavily dominated the list of major constituents of the Saudi species, and, out of the list of 73 compounds, only 11 compounds were present in more than 1% of the total phytochemical constituents (see Table 1). The major compounds in the Saudi species were *α*-bisabolol (27.8%), *γ*-gurjunenepoxide (21.7%), (*E*, *E*)-*α*-farnesene (16.3%), *cis*-spiroether (7.5%), (*E*)-*β*-farnesene (2.7%), diepicedrene-1-oxide (2.0%), artemesia ketone (1.8%), palmitic acid (1.6%), (*Z*, *E*)-*α*-farnesene (1.4%), (*Z*)-*β*-farnesene (1.3%), phytol (1.2%), and germacrene D (1.0%). The remaining 62 compounds were only present in <1% amounts of the total constituents of Saudi *M. aurea*. Notably, among the total 118 phytoconstituents present in the Jordanian *M. aurea*, only 7 compounds were present in relatively large quantities (>1% of the total compounds), while the remaining 111 compounds were present in minute quantities. Almost all the major compounds were same in both species, except *α*-bisabolol, which was completely absent in the Jordanian species (Figure 1). The chemical structures of lead compounds identified from both Saudi and Jordanian essential oils of *M. aurea* are given in Figure 2. Indeed, the major difference between the two species is the presence of *α*-bisabolol, which can be exploited for the chemotaxonomic identification of the Saudi *M. aurea* [36].

**Figure 3:**
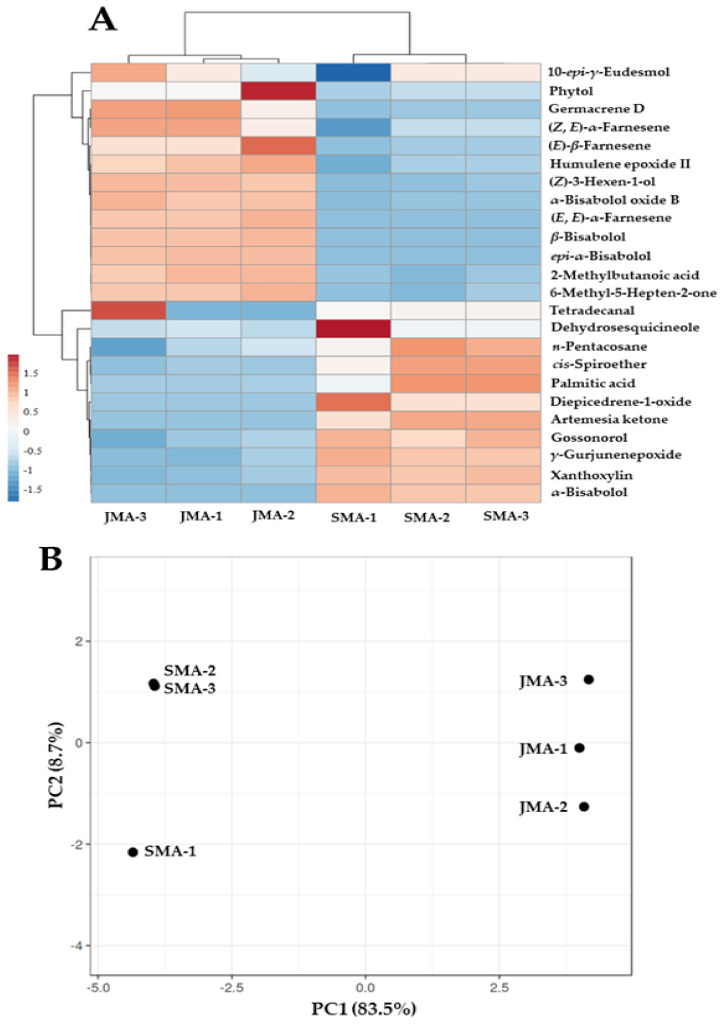
(**A**) Dendrogram heatmaps showing comparative quantities of various essential oil components detected in Saudi (SMA) and Jordanian (JMA) *M. aurea* samples. Twenty-four components with a content of more than or equal to 0.5% of the total oil composition are used for analysis. (**B**) PCA of essential oil composition of Saudi and Jordanian samples, showing a distinct clustering of the two samples.

The heatmap and dendrograms (Figure 3) were created using a total of twenty-four different types of phytomolecules found in each oil sample in variable quantities. For this analysis, only the phytomolecules with a quantity of 0.5% or more were included. The data obtained from these phytomolecules (>0.5%) revealed that the Jordanian and Saudi samples clearly form distinct clusters, which further confirmed our initial analysis as detailed earlier, i.e., the samples obtained from Jordanian and Saudi EOs exhibit distinct essential oil profiles. Saudi samples are marked by the higher content of oxygenated sesquiterpenes, including α-bisabolol, γ-gurjunenepoxide, gossonorol, and dehydrosesquicineole (Table 2), whereas Jordanian samples have a distinctly higher content of sesquiterpene hydrocarbons, such as (*E*, *E*)-α-farnesene, (*E*)-*β*-farnesene, and (*Z*, *E*)-α-farnesene. The biplot for PC1 and PC2 further confirmed that the essential oil profiles associated with Jordanian EOs are quite distinct from those of Saudi EOs (Figure 3B). The PCA analysis and the dendrogram therefore confirm that the Saudi samples are quite different than the Jordanian samples. A rigorous analysis based on a higher number of samples is required in future.

*α*-Bisabolol belongs to the class of unsaturated monocyclic sesquiterpene alcohols, and is widely considered as one of the “most-used herbal constituents” globally [37]. So far, a broad range of biological and therapeutic properties of *α*-bisabolol have been reported, including anti-oxidative and anti-cancer properties, for the treatment of inflammatory and metabolic disorders and neurodegenerative diseases [38]. Four different stereoisomers of *α*-bisabolol possibly exist in nature, i.e., (–)-*α*-bisabolol (known as levomenol), (–)-*epi*-*α*-bisabolol, (+)-*α*-bisabolol, and (+)-*epi*-*α*-bisabolol [39]. Bisabolol is a low-density (0.93 in 20 °C) and pale-yellowish liquid, which can be easily oxidized to produce two bisabolol oxides (bisabolol-oxide A and B) [40]. Notably, this compound is not present in all the species of *M. aurea* growing in different regions of the world; indeed, this substance is specific to the plants such as *M. aurea* and other plants of genus *Matricaria* which are found under harsh climatic conditions, such as very humid and hot summers with annual precipitation (ranging from 235 to 455 mm), e.g., the Persian Gulf with mild winters and the hot regions of Saudi Arabia, as specifically found in this study [41]. For instance, this compound is not present in the Jordanian *M. aurea* as revealed in the present study; in addition, it is also not found in the Tunisian and Indian species [42,43]. However, other than *M. aurea*, *α*-bisabolol can also be found in other plant species including *M. recutita*, *Salvia runcinata*, *Silene stenophylla*, *Vanillosmopsis pohlii*, *Vernonia arborea*, *Myoporum crassifolium*, and *Eremanthus erythropappus*. Indeed *M. chamomilla* is considered one of the major sources of this compound and consists of up to 50.0% *α*-bisabolol [44]. When the overall components of the Saudi and Jordanian EOs were compared to the same species of plants from other regions, it was clearly revealed that the major components were completely different from each other (cf. Table 2).

**Table 2:** Major components of *M. aurea* from different parts of the world.

No.	Country	City	Major Components (%)	Reference
1.	Tunisia	Sousse	1,5 Bis (dicyclohexylphosphino)-pentane (4.0–44.7), 2-Ethoxy-6-ethyl-4,4,5- trimethyl-1,3-dioxa-4-sila-2 boracyclohex-5-ene (6.5–38.0), octahydrocoumarin 5,7-dimethyl (0–19.2), pentadecanoic acid (0–16.0), lauric acid (0–13.7), (2,5-Bis1,1-8 dimethyleth)thiophene (0–11.0), *n*-eicosanol (0–10.0), *n*-eicosane (0–6.6).	[43]
2.	Saudi Arabia	Alkharj	Bisabolol oxide A (64.8), *n*-nonadecane (6.7), 2R,3R, ALL-E)-2,3-Epoxy-2,6,10,14-tetramethyl-16-(phenylthio) hexadeca-6,10,14-triene (5.8), *trans*-*β*-farnesene (3.0), 1-fluorododecane (2.1), *β*-bisabolene (1.9).	[45]
3.	Jordan	Amman	(*E*, *E*)-*α*-Farnesene (50.2), *γ-*gurjunenepoxide (8.5), (*E*)-*β*-farnesene (8.1), *(Z*, *E)-α-*farnesene (4.4), *cis-*spiroether (3.9).	Present study
4.	Saudi Arabia	Riyadh	*α-*Bisabolol (27.8), *γ-*gurjunenepoxide (21.7), (*E*, *E*)-*α*-farnesene (16.3), *cis-*spiroether (7.5), (*E*)-*β*-farnesene (2.7), *(Z*, *E)-α-*farnesene (1.4).	Present study

In the present study, we have, for the first time, discovered the presence of α-bisabolol in the *M. aurea* of Saudi Arabia, which is typically known to be present within the genus *Matricaria* of the family Asteraceae, but is only found in plants that grow under specific climatic conditions [42]. Particularly, to the best of our knowledge, to date *α*-bisabolol has not been found in the *M. aurea* plants of other regions, including Jordan, Tunisia, and India [42,43]. Therefore, it can be effectively used as a valuable marker to support the taxonomic classification of *M. aurea* species. Furthermore, several other derivatives of bisabolol were identified in the studied plants, such as *β*-bisabolol, *epi*-*α*-bisabolol, *α*-bisabolol oxide A, and *α*-bisabolol oxide B, which can be of vital importance as chemotaxonomic markers of the genus *Matricaria*. Notably, none of the derivatives of bisabolol were found in the Jordanian species. Therefore, compounds 101, 105, 106, 107, and 113 from Table 1, which are reported for the first time in the *M. aurea* plant of Saudi Arabia, can be used as further chemical markers to distinguish Saudi *M. aurea* from other *Matricaria* species growing in other regions of the world [42,43,45]. Since the *M. aurea* plant is widely applied in Saudi Arabia for various medicinal purposes, the biological/toxicological profile of the phytoconstituents of *M. aurea* may offer useful information. In particular, the isolation of *α*-bisabolol and its in vitro and in silico studies, which we plan to perform in our future research, may offer valuable information.

## 3. Materials and Methods

### 3.1. Plant Material

Whole aerial parts of *M. aurea* grown wildly in two different agro-climatic conditions, namely Riyadh, Saudi Arabia and Amman, Jordan, were procured in the month of March. Fresh plant materials were taxonomically identified at the herbarium division of King Saud University (Riyadh, Saudi Arabia) and then processed further for the isolation of essential oils.

### 3.2. Extraction of M. aurea Essential Oils

Firstly, the fresh whole aerial parts of the procured *M. aurea* from Saudi Arabia and Jordan were chopped into small pieces and subjected to hydro-distillation in a conventional Clevenger apparatus for three hours as described previously [31]. After hydro-distillation of Saudi and Jordanian *M. aurea* plant materials, light-yellow-colored oils with yields of 0.03 and 0.05% on a fresh weight basis, respectively, were obtained. These essential oils obtained from the aerial parts of the *M. aurea* were dried over anhydrous Na_2_SO_4_ and stored at 4 °C until they were analyzed.

### 3.3. GC and GC–MS Analysis of M. aurea Essential Oils

In order to identify the chemical constituents of the Saudi and Jordanian *M. aurea* essential oils, volatile oils were dissolved in diethyl ether and subjected to GC–FID and GC–MS analyses. The GC analysis was carried out employing two different stationary phase columns, i.e., a nonpolar (HP-5MS) and a polar (DB-Wax) column using the same method as described previously [30]. The detailed methodology is given in Supplementary Materials (Scheme S1). The identified constituents from the Saudi and Jordanian *M. aurea* essential oils and their relative percentages are given in Table 1 and the identified constituents are listed according to their elution order on the HP-5MS column.

### 3.4. Calculation of Linear Retention Indices (LRIs)

LRI values of chemical constituents of Saudi and Jordanian *M. aurea* essential oils were determined employing previously reported procedures [30], and LRI values of each component are listed in Table 1. The detailed methodology is provided in Supplementary Materials (Scheme S2).

### 3.5. Identification of Volatile Chemical Components

**Figure 4:**
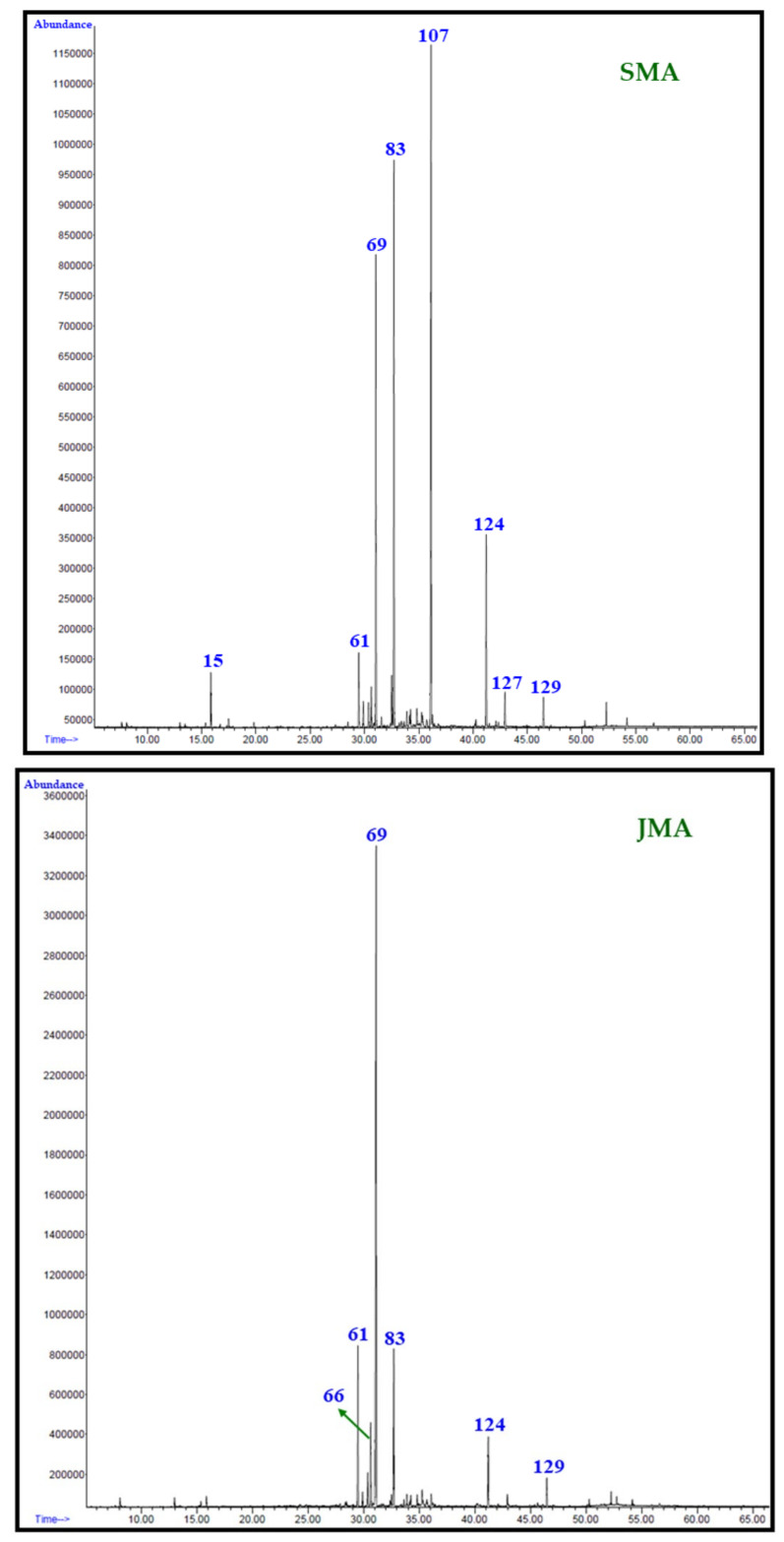
Total ion chromatogram (TIC) of Saudi (SMA) and Jordanian (JMA) *M. aurea* essential oils.

Identification of the chemical constituents of Saudi and Jordanian *M. aurea* essential oils was achieved through analysis of both oils on two different stationary phase columns, namely the HP-5MS and DB-Wax columns, as described previously [30]. The detailed methodology for the identification of chemical constituents of Saudi and Jordanian *M. aurea* essential oils is provided in detail in Supplementary Materials (Scheme S3) [32,33,34,35]. GC chromatograms of the analysis of both essential oils on an HP-5MS column are given in Figure 4.

### 3.6. Statistical Analysis

Heatmap and Principal Component Analysis (PCA) analyses were performed to evaluate the difference in the chemical constituents of the EOs of Saudi and Jordanian *M. aurea*. For the purpose of statistical analysis, each sample of EO was injected three times into a GC to obtain the standard deviation of the contents of the oil components. The data sets of the Saudi EO were named SMA-1, SMA-2, and SMA-3, while the Jordanian oil was referred to as JMA-1, JMA-2, and JMA-3. The data obtained were further used for heatmap and PCA analyses. An overall clustering of the six samples based on twenty-four different phytomolecules showing a content of more than or equal to 0.5% was carried out by calculating dendrograms, heat maps, and PCA using the web-based tool Clustvis [46]. To visualize the relationship between the Saudi and Jordanian EOs, Clustvis-based R tools such as ggplot2, pheat, and pcaplot were used.

## 4. Conclusions

In this study, we explored the phytoconstituents of the essential oils of *M. aurea* obtained from different countries, i.e., Saudi Arabia and Jordan. The detailed chemical characterization of the volatile compounds of collected *M. aurea* plants was performed and the results of both the plants were extensively compared. The essential oils of *M. aurea* from Saudi Arabia exhibited a significant difference in their chemical compositions when compared to its counterpart collected from Jordan. Here, the presence of *α*-bisabolol was revealed as a major component (~27%) of Saudi *M. aurea*, which has so far not been found in the same plant from other regions, including Jordan, India, and Tunisia. By comparison, the Jordanian *M. aurea* consisted of *γ*-gurjunenepoxide (~22%) as its major constituent, which is also present in the Saudi plant, but in a relatively small quantity (~9%). Furthermore, the studied plants also contain *(E, E)-α*-farnesene (16.3%), *cis*-spiroether (7.5%), *(E)-β*-farnesene (2.7%), diepicedrene-1-oxide (2.0%), artemesia ketone (1.8%), palmitic acid (1.6%), *(Z*, *E)-α-*farnesene (1.4%), *(Z)-β-*farnesene (1.3%), phytol (1.2%), and germacrene *D* (1.0%). Therefore, *α*-bisabolol can be used as a valuable marker to support the taxonomic classification of *M. aurea* species. Furthermore, this study also reaffirms the same plant having different origins can have different phytochemical profiles which can be effectively used for the purpose of chemotaxonomic classifications. This was also confirmed by dendrograms and PCA analysis. Furthermore, it is important to mention that, in the present study, detailed chemical investigation of *M. aurea* revealed the presence of various antimicrobial agents. In particular, the presence of *α*-bisabolol as a major component in the Saudi *M. aurea* oil may provide great opportunity for the isolation of bioactive compounds, which could be used as potential candidates in the chemotherapy of infectious diseases.

## Figures and Tables

**Table 1:** Percentage composition of essential oils from aerial parts of *M. aurea* grown in Saudi Arabia and Jordon.

No.	Compounds *	M.F.	R.T. (min.)	LRI_Lit_	LRI_Exp_^a^	LRI_Exp_^p^	SMA (%) ^b^	JMA (%) ^b^
1	(*E*)-3-Hexen-1-ol	C_6_H_12_O	7.958	844	849	1370	-	t
2	(*Z*)-3-Hexen-1-ol	C_6_H_12_O	8.081	850	853	1389	0.2	0.5
3	2-Methylbutanoic acid	C_5_H_10_O_2_	8.173	832	855	-	0.2	0.5
4	1-Hexanol	C_6_H_14_O	8.533	863	867	1359	-	0.1
5	*n*-Nonane	C_9_H_20_	9.711	900	900	900	-	t
6	6-Methyl-5-Hepten-2-one	C_8_H_14_O	12.994	981	987	1338	0.2	0.5
7	2-Pentylfuran	C_9_H_14_O	13.158	984	991	1232	-	0.1
8	Yomogi alcohol	C_10_H_18_O	13.461	999	999	1402	0.1	0.1
9	*α*-Terpinene	C_10_H_16_	14.143	1014	1017	-	-	0.1
10	Limonene	C_10_H_16_	14.62	1024	1029	1197	0.1	-
11	(*Z*)-*β*-Ocimene	C_10_H_16_	14.957	1032	1038	1235	-	t
12	Benzeneacetaldehyde	C_8_H_8_O	15.187	1036	1044	1642	-	0.1
13	(*E*)-*β*-Ocimene	C_10_H_16_	15.356	1044	1049	1252	0.2	0.3
14	*γ*-Terpinene	C_10_H_16_	15.573	1054	1055	-	-	t
15	**Artemesia ketone**	**C_10_H_16_O**	**15.851**	**1056**	**1062**	**1350**	**1.8 ± 0.24**	0.5
16	*cis*-Linalool oxide	C_10_H_18_O_2_	16.157	1067	1073	1447	-	0.1
17	Artemesia alcohol	C_10_H_18_O	16.7	1080	1084	1511	-	t
18	Linalool	C_10_H_18_O	17.312	1095	1100	1552	0.1	0.1
19	Nonanal	C_9_H_18_O	17.477	1100	1105	1395	0.3	0.1
20	Isoamyl isovalerate	C_10_H_20_O_2_	17.655	-	1109	-	-	t
21	*β*-Thujone	C_10_H_16_O	17.873	1112	1115	-	0.1	-
22	Menthone	C_10_H_18_O	19.294	1148	1154	1470	-	0.1
23	Pinocarvone	C_10_H_14_O	19.538	1160	1161	-	t	-
24	Lavandulol	C_10_H_18_O	19.807	1165	1168	1682	0.2	0.1
25	Naphthalene	C_10_H_8_	20.425	1178	1185	1740	-	0.1
26	*n*-Dodecane	C_12_H_26_	20.95	1200	1200	1200	-	0.1
27	*n*-Decanal	C_10_H_20_O	21.182	1201	1206	1500	0.1	-
28	Hexyl 2-methylbutyrate	C_11_H_22_O_2_	22.254	-	1237	1430	0.1	-
29	Carvone	C_10_H_14_O	22.323	1239	1239	-	t	0.1
30	Geraniol	C_10_H_18_O	22.869	1249	1255	-	-	0.1
31	Benzyl propanoate	C_10_H_12_O_2_	23.066	1257	1260	1796	0.1	0.1
32	*trans*-2-Decenal	C_10_H_18_O	23.292	1260	1267	1639	-	0.1
33	Geranial	C_10_H_16_O	23.452	1264	1272	1735	-	0.1
34	Methyl 3-phenylpropanoate	C_10_H_12_O_2_	23.69	-	1279	1857	-	0.1
35	*p*-Ethylacetophenone	C_10_H_12_O	24.071	1279	1282	-	-	0.1
36	Thymol	C_10_H_14_O	24.23	1289	1294	-	-	t
37	Perilla alcohol	C_10_H_16_O	24.276	1294	1296	2001	-	0.2
38	*n*-Tridecane	C_13_H_28_	24.392	1300	1300	1300	-	t
39	Carvacrol	C_10_H_14_O	24.675	1298	1304	2215	-	0.1
40	*n*-Undecanal	C_11_H_22_O	24.762	1305	1310	1607	-	0.1
41	2-Methylnaphthalene	C_11_H_10_	24.851	-	1313	-	-	0.1
42	(2*E*, 4*E*)-Decadienal	C_10_H_16_O	24.991	1315	1317	1807	0.2	0.1
43	Myrteny acetate	C_15_H_24_	25.164	1324	1323	1691	-	0.1
44	*δ*-Elemene	C_15_H_24_	25.758	1335	1341	1472	t	t
45	Piperitenone	C_10_H_14_O	25.799	1340	1343	-	-	t
46	Eugenol	C_10_H_12_O_2_	26.487	1356	1361	2164	-	0.1
47	*cis*-Carvyl acetate	C_12_H_18_O_2_	26.537	1365	1365	-	t	-
48	Biphenyl	C_12_H_10_	27.061	-	1381		-	t
49	*β*-Maaliene	C_15_H_24_	27.186	-	1385	1524	-	t
50	Benzyl isovalerate	C_12_H_16_O_2_	27.321	-	1389		0.1	0.1
51	*α*-Isocomene	C_15_H_24_	27.316	1387	1390	-	-	t
52	*β*-Cubebene	C_15_H_24_	27.517	1387	1395	-	-	0.1
53	Tetradecane	C_14_H_30_	27.65	1400	1400	1400	t	0.1
54	2, 6-Dimethylnaphthalene	C_12_H_12_	27.868	-	1406	-	-	0.2
55	*cis*-*α*-Bergamotene	C_15_H_24_	27.931	1411	1415		0.1	t
56	*β*-Caryophyllene	C_15_H_24_	28.471	1417	1426	1599	0.2	0.3
57	*trans*-*α*-Ionone	C_13_H_20_O	28.598	1428	1430	-	-	0.1
58	*β*-Gurjunene	C_15_H_24_	28.753	1431	1435	1595	-	0.1
59	Aromadendrene	C_15_H_24_	28.894	1439	1440	1624	-	0.1
60	(*Z*)-*β*-Farnesene	C_15_H_24_	29.063	1440	1445	1654	0.2	0.1
61	**(*E*)-*β*-Farnesene**	**C_15_H_24_**	**29.47**	**1454**	**1459**	**1668**	**2.7 ± 0.30**	**8.1 ± 2.62**
62	Dehydrosesquicineole	C_15_H_24_O	29.896	-	1473	1721	0.9	0.8
63	*α*-Curcumene	C_15_H_22_	30.229	1479	1483	1776	-	0.1
64	**Germacrene D**	**C_15_H_24_**	**30.364**	**1484**	**1487**	**1712**	**1.0 ± 0.02**	**1.9 ± 0.30**
65	*trans*-*β*-Ionone	C_13_H_20_O	30.533	1487	1493	1944	-	0.1
66	**(*Z*, *E*)-*α*-Farnesene**	**C_15_H_24_**	**30.622**	**-**	**1496**	**1728**	**1.4 ± 0.85**	**4.4 ± 1.03**
67	Bicyclogermacrene	C_15_H_24_	30.833	1500	1503	1737	0.2	0.2
68	*α*-Muurolene	C_15_H_24_	30.906	1500	1506	1724	0.2	0.1
69	**(*E*, *E*)-*α*-Farnesene**	**C_15_H_24_**	**31.116**	**1505**	**1513**	**1752**	**16.3 ± 0.02**	**50.2 ± 3.25**
70	*γ*-Cadinene	C_15_H_24_	31.289	1513	1517	-	0.1	-
71	7-*epi*-*α*-Selinene	C_13_H_14_	31.293	1520	1519	1769	-	0.1
72	*β*-Sesquiphellanderene	C_15_H_24_	31.561	1521	1528	1773	0.4	0.2
73	(*Z*, *E*)-Matricaria ester	C_14_H_12_O_4_	31.594	-	1530	-	-	0.2
74	*trans*-*γ*-Bisabolene	C_15_H_24_	31.732	1529	1534	-	-	0.1
75	*α*-Cadinene	C_15_H_26_	31.816	1537	1537	-	-	0.1
76	(*E*, *E*)-Matricaria ester	C_14_H_12_O_4_	31.97	-	1543	-	0.3	0.1
77	*α*-Calacorene	C_15_H_20_	32.047	1544	1545	1922	-	0.1
78	Elemol	C_15_H_26_O	32.214	1548	1551	2077	-	0.1
79	Elemicin	C_12_H_16_O_3_	32.310	1555	1554	2231	-	0.1
80	Sesquirosefuran	C_15_H_22_O	32.391	-	1557	1896	0.2	0.4
81	**Diepicedrene-1-oxide**	**C_15_H_24_O**	**32.501**	**-**	**1561**	**1942**	**2.0 ± 0.63**	0.6
82	*trans*-Nerolidol	C_15_H_26_O	32.587	1561	1564		-	0.1
83	***γ*-Gurjunenepoxide**	**C_15_H_24_O**	**32.71**	**-**	**1568**	**1966**	**21.7 ± 1.01**	**8.5 ± 2.23**
84	Caryophyllenyl alcohol	C_15_H_26_O	32.891	1570	1574	2051	0.1	-
85	Spathulenol	C_15_H_24_O	33.207	1577	1585	2131	0.2	0.1
86	Caryophyllene oxide	C_15_H_24_O	33.395	1582	1592	1990	0.3	0.2
87	*n*-Hexadecane	C_16_H_34_	33.632	1600	1600	-	-	t
88	Sesquithuriferol	C_15_H_26_O	33.683	1604	1601	-	0.3	0.4
89	Geranyl isovalerate	C_15_H_26_O_2_	33.796	1606	1605	1905	0.1	0.1
90	Humulene epoxide II	C_15_H_24_O	33.895	1608	1609	2047	0.6	0.7
91	Tetradecanal	C_14_H_28_O	34.049	1611	1615	1925	0.5	0.1
92	*epi*-Cedrol	C_15_H_26_O	34.145	1618	1618	2148	-	0.3
93	10-*epi*-*γ*-Eudesmol	C_15_H_26_O	34.24	1622	1622	2106	0.7	0.7
94	*γ*-Eudesmol	C_15_H_26_O	34.502	1630	1631	2172	0.2	0.2
95	*α*-Acorenol	C_15_H_26_O	34.514	1632	1633	2163	0.1	0.1
96	Gossonorol	C_15_H_22_O	34.821	1636	1643	2310	0.9	0.7
97	*τ*-Muurolol	C_15_H_26_O	34.96	1640	1647	-	t	-
98	*α*-Muurolol	C_15_H_26_O	34.976	1644	1649	2187	0.2	0.3
99	*β*-Eudesmol	C_15_H_26_O	35.058	1649	1652	2238	0.3	0.1
100	*α*-Eudesmol	C_15_H_26_O	35.171	1652	1656		0.1	0.1
101	*α*-Bisabolol oxide B	C_15_H_26_O_2_	35.261	1656	1659	2142	0.6	0.9
102	Xanthoxylin	C_10_H_12_O_4_	35.349	-	1662		0.6	0.4
103	Intermedeol	C_15_H_26_O	35.411	1665	1664	-	-	0.1
104	Tridecanoic acid	C_13_H_26_O_2_	35.6	-	1671	-	-	0.2
105	*β*-Bisabolol	C_15_H_26_O	35.678	1674	1674	2140	-	0.6
106	*epi*-*α*-Bisabolol	C_15_H_26_O	36.071	1683	1688	2115	-	0.9
107	***α*-Bisabolol**	**C_15_H_26_O**	**36.128**	**1685**	**1691**	**2223**	**27.8 ± 1.37**	**-**
108	Geranyl tiglate	C_15_H_26_O	36.233	1696	1694	2097	-	0.3
109	(*Z*, *Z*)-Farnesol	C_15_H_24_O_2_	36.414	1698	1700	2322	0.2	0.1
110	Pentadecanal	C_15_H_30_O	36.786	-	1715	2043	0.2	0.1
111	(*Z*, *E*)-Farnesol	C_15_H_26_O	37.023	1722	1724	2365	t	t
112	*β*-Farnesol	C_15_H_26_O	37.577	1742	1745		t	-
113	*α*-Bisabolol oxide A	C_15_H_26_O_2_	37.908	1748	1758	2429	t	-
114	Benzyl benzoate	C_14_H_12_O_2_	37.992	1759	1761	2607	0.1	-
115	Tetradecanoic acid	C_14_H_28_O_2_	38.086	-	1765		0.1	-
116	Gurjunazulen	C_15_H_18_	38.236	-	1770		0.1	-
117	3, 4′-Dimethylbiphenyl	C_14_H_14_	38.387	-	1776		0.1	-
118	8-Cedren-13-ol acetate	C_17_H_26_O	38.701	1788	1788		-	t
119	Octadecene	C_18_H_36_	38.807	1789	1792		-	0.1
120	Farnesyl acetate	C_17_H_28_O_2_	39.989	1845	1839	2257	-	t
121	Phytone	C_18_H_36_O	40.166	-	1846	2152	0.3	0.3
122	(*Z*, *Z*)-Farnesyl acetone	C_18_H_30_O	40.261	1860	1850		0.1	0.2
123	Pentadecanoic acid	C_15_H_30_O_2_	40.794	-	1871	-	-	t
124	***cis*-Spiroether**	**C_13_H_12_O**	**41.215**	**1879**	**1888**	**-**	**7.5 ± 1.23**	**3.9 ± 0.40**
125	*trans*-Spiroether	C_13_H_12_O	41.499	1890	1899	-	0.2	0.1
126	Methyl hexadecanoate	C_17_H_34_O_2_	42.107	1921	1925	2204	0.2	0.2
**127**	**Palmitic acid**	**C_16_H_32_O**_2_	**42.939**	**1959**	**1960**	**-**	**1.6 ± 0.40**	0.7
128	Methyl linoleate	C_19_H_34_O_2_	46.105	2095	2093	-	-	0.2
129	**Phytol**	**C_20_H_40_O**	**46.999**	**1942**	**2129**	**2620**	**1.2 ± 0.06**	**1.6 ± 0.72**
130	Linoleic acid	C_17_H_30_O_2_	47.034	2132	2132	-	-	0.1
131	Oleic acid	C_18_H_34_O_2_	47.156	2141	2137	-	-	0.1
132	*α*-Linolenic acid	C_18_H_30_O_2_	47.166	-	2138	-	0.1	-
133	*cis*-13-Octadecen-1-yl-acetate	C_20_H_38_O_2_	48.523	-	2194		-	0.1
134	*n*-Tricosane	C_23_H_48_	51.101	2300	2300	2300	-	t
135	*n*-Pentacosane	C_25_H_52_	55.901	2500	2500	2500	0.7	0.6
*Monoterpenes hydrocarbons*							0.3	0.4
*Oxygenated monoterpenes*							2.3	1.7
*Sesquiterpene hydrocarbons*							24.0	66.4
*Oxygenated sesquiterpenes*							58.2	18.46
*Aliphatic hydrocarbons*							0.7	0.9
*Oxygenated aliphatic hydrocarbons*							4.0	3.9
*Diterpenoids*							1.2	1.6
*Aromatics*							1.0	1.3
*Polyacetylenic*							7.7	4.0
*Other components*							14.6	11.7
**Total identified**							**98.3**	**98.7**

* Components are recorded as per their order of elution from an HP-5MS column; compounds higher than 1.0% are highlighted in boldface; LRI_Lit_ = linear retention index from the literature [32,33,34,35]; LRI_Exp_^a^ = linear retention index computed with reference to the *n*-alkanes mixture (C8–C31) on an HP-5MS column; LRI_Exp_^p^ = linear retention index computed with reference to the *n*-alkanes mixture (C8-C31) on a DB-Wax column; SMA = Saudi *M. aurea* oil; JMA = Jordanian *M. aurea* oil; ^b^ = Mean percentage calculated from a flame ionization detector (FID).

## Data Availability

Data contained within the article.

## Supplementary Materials

The following supporting information can be downloaded at: https://www.mdpi.com/article/10.3390/plants12203553/s1, Scheme S1: Gas Chromatography (GC) and Gas Chromatography–Mass Spectrometry (GC-MS) Analysis of *M. aurea* Essential oils; Scheme S2: Linear Retention Indices (LRIs); Scheme S3: Identification of Volatile Components.
