# Supplementary material for: Chemical Characterization and Chemotaxonomic Significance of Essential Oil Constituents of Matricaria aurea Grown in Two Different Agro-Climatic Conditions
Source: Plants (Basel). 2023 Oct 12;12(20):3553. doi: 10.3390/plants12203553 (PMC10610148; doi:10.3390/plants12203553)
Supplement: Supplementary file 1 [file plants-12-03553-s001.zip › plants-2607912-supplementary.pdf]

*Supplementary File*

# Chemical Characterization and Chemotaxonomic Significance of Essential Oil Constituents of *Matricaria aurea* Grown in Two Different Agro-Climatic Conditions


**Merajuddin Khan[1] *, Mujeeb Khan[1], Eman Alshareef[2], Shatha Ibrahim Alaqeel[2], and Hamad Z. Alkhathlan[1]***

1 Department of Chemistry, College of Science, King Saud University, P.O. Box 2455, Riyadh 11451, Saudi Arabia; kmujeeb@ksu.edu.sa

2 Department of Chemistry, College of Science, King Saud University (034), Riyadh 11495, Saudi Arabia; 438203607@student.ksu.edu.sa (E.A.); shalaqeel@ksu.edu.sa (S.I.A.)

* Correspondence: mkhan3@ksu.edu.sa; khathlan@ksu.edu.sa; Tel.: +966-11-4675910


**Scheme S1. Gas Chromatography (GC) and Gas Chromatography–Mass Spectrometry (GC-MS) Analysis of *M. aurea* Essential oils**

The volatile oils were analyzed using a GC–MS and GC–FID equipped with two columns, one of which was polar (DB-Wax) and the other was nonpolar (HP-5MS). GC–MS was performed on an Agilent single-quadrupole mass spectrometer with an inert mass selective detector (MSD-5975C detector, Agilent Technologies, USA) coupled directly to an Agilent 7890A gas chromatograph which was equipped with a split–splitless injector, a quickswap assembly, an Agilent model 7693 autosampler, and a HP-5MS fused silica capillary column (5% phenyl 95% dimethylpolysiloxane, 30 m × 0.25 mm i.d., film thickness 0.25 μm, Agilent Technologies, USA). Supplementary analyses were performed on a DB-Wax fused silica capillary column (polyethylene glycol, 30 m × 0.25 mm i.d., film thickness 0.25 μm, Agilent Technologies, USA). The HP-5MS column was operated using an injector temperature of 250 °C and the following oven temperature profile: an isothermal hold at 50 °C for 4 min, followed by a ramp of 4 °C/min to 220 °C, an isothermal hold for 2 min, a second ramp to 280 °C at 20 °C/min, and finally an isothermal hold for 15 min. Conversely, the DB-Wax column was operated using an injector temperature of 250 °C and the following oven temperature profile: an isothermal hold at 40 °C for 4 min, followed by a ramp of 4 °C/min to 220 °C and an isothermal hold for 10 min.

Approximately 0.2 μl of each sample diluted in diethyl ether was injected using the split injection mode; the split flow ratio was 10:1. The helium carrier gas was flowed at 1 ml/min. The GC–TIC profiles and mass spectra were obtained using the ChemStation data analysis software, version E-02.00.493 (Agilent). All mass spectra were acquired in the EI mode (scan range of m/z 45–600 and ionization energy of 70 eV). The temperatures of the electronic-impact ion source and the MS quadrupole were 230 °C and 150 °C, respectively. The MSD transfer line was maintained at 280 °C for

both polar and nonpolar analysis. The GC analysis was performed on an Agilent GC-7890A dual-channel gas chromatograph (Agilent Technologies, USA) equipped with FID using both polar (DB-Wax) and nonpolar (HP-5MS) columns under the same conditions as described above. The detector temperature was maintained at 300 °C for both polar and nonpolar analyses. The relative composition of the oil components was calculated on the basis of the GC–FID peak areas measured using the HP-5 MS column without using a correction factor. Results are reported in Table 1 according to their elution order on the HP-5MS column.

**Scheme S2**. **Linear Retention Indices (LRIs)**

A mixture of a continuous series of straight-chain hydrocarbons, C8-C31 (C8-C20, 04070, Sigma-Aldrich, USA and C20-C31, S23747, AccuStandard, USA) was injected into both polar (DB-Wax) and nonpolar (HP-5MS) columns under the same conditions described in scheme S1 for the oil samples to obtain the linear retention indices (LRIs) (also referred to as linear temperature programmed retention indices (LTPRI)) of the oil constituents provided in Table 1. The LRIs were computed using van den Dool and Kratz's equation.

**Scheme S3. Identification of Volatile Components**

The identification of different phytocomponents of the essential oils of *M. aurea* was performed by matching their mass spectra with the library entries of mass spectra databases (WILEY 9th edition, NIST-14 MS library version 2.2, and Adams and Flavor libraries), as well as by comparing their mass spectra and linear retention indices (LRIs) with published data on both polar and nonpolar columns [1-4] and the co-injection of authentic standards available in our laboratory.

## References


1. Adams, R., Identification of essential oil components by gas chromatography/mass spectrometry. *Identification of essential oil components by gas chromatography/mass spectrometry.* **2007**, (Ed. 4).
2. Acree, T.; Arn, H., Flavornet. Cornell University, NYSAES. New York. 2012.
3. Linstrom, P.; Mallard, W., NIST Chemistry WebBook, NIST Standard Reference Database Number 69, June 2005. *National Institute of Standards and Technology, Gaithersburg MD* **2005,** 20899.
4. Babushok, V.; Linstrom, P.; Zenkevich, I., Retention indices for frequently reported compounds of plant essential oils. *J. Phys. Chem. Ref. Data* **2011,** 40, (4).
